# Muscle Satellite Cells: Exploring the Basic Biology to Rule Them

**DOI:** 10.1155/2016/1078686

**Published:** 2016-03-03

**Authors:** Camila F. Almeida, Stephanie A. Fernandes, Antonio F. Ribeiro Junior, Oswaldo Keith Okamoto, Mariz Vainzof

**Affiliations:** Human Genome and Stem Cell Research Center, IBUSP, Rua do Matão 106, Cidade Universitária, 5508-900 São Paulo, SP, Brazil

## Abstract

Adult skeletal muscle is a postmitotic tissue with an enormous capacity to regenerate upon injury. This is accomplished by resident stem cells, named satellite cells, which were identified more than 50 years ago. Since their discovery, many researchers have been concentrating efforts to answer questions about their origin and role in muscle development, the way they contribute to muscle regeneration, and their potential to cell-based therapies. Satellite cells are maintained in a quiescent state and upon requirement are activated, proliferating, and fusing with other cells to form or repair myofibers. In addition, they are able to self-renew and replenish the stem pool. Every phase of satellite cell activity is highly regulated and orchestrated by many molecules and signaling pathways; the elucidation of players and mechanisms involved in satellite cell biology is of extreme importance, being the first step to expose the crucial points that could be modulated to extract the optimal response from these cells in therapeutic strategies. Here, we review the basic aspects about satellite cells biology and briefly discuss recent findings about therapeutic attempts, trying to raise questions about how basic biology could provide a solid scaffold to more successful use of these cells in clinics.

## 1. Introduction

Skeletal muscle is a postmitotic tissue that has a high regenerative potential. This feature is mainly due to satellite cells (SCs), which form a reservoir of precursor cells that are responsible for its after-birth growth and also for the response to injuries, either by exercise or by disease [1]. Their amounts in the adult muscle could vary between 3 and 11% of the myonuclei, depending upon which species are being analyzed. In mice, the amount of SCs drops from 32% in neonates to 5% in adults [2, 3]. These cells are strictly associated with the sarcolemma, residing between the membrane and the basal lamina [4], becoming associated with the muscle fiber before the formation of its surrounding lamina [3].

These cells are easily identified by their location and morphology. However, efficient ways to obtain these cells involve the use of several markers that characterize this cell type, the transcription factor Pax7 being the most remarkable one [5]. Even though they are well studied and recognized, the SC population is highly heterogeneous [6].

Although quiescent in normal adult muscles, these cells can be activated by specific signals when a muscle injury occurs. Upon activation these cells undergo asymmetric division, by which they could form cells that either are capable of self-renewing or can enter the myogenic pathway and differentiate to restore the muscle [7–9]. Nonetheless, in diseases characterized by relentless degeneration, like muscular dystrophies, the satellite cells are constantly activated, which eventually leads to depletion of the SC pool and consequent failure of the regeneration process [10]. Currently, there is no effective treatment for muscle degenerative diseases; thus, many researchers are focusing on stem cell-based therapies. However, to date, most attempts are limited to animal models and former clinical trials have failed.

In this review, we summarize recent findings about the basic biology of muscle-specific stem cells and discuss possible new avenues to more effective and feasible therapeutic approaches to muscle wasting disorders, mainly muscular dystrophies.

## 2. Origin of Satellite Cells in the Muscle Development 

In the embryo, mesoderm structures called somites are formed and skeletal muscles are derived from a specific region, the dermomyotome [11]. In this step the first muscle fibers are formed and additional fibers are added afterwards using the former as a template [12, 13]. In the final period of embryogenesis, muscle progenitors start to proliferate vastly until they arrive in a state in which the number of nuclei is maintained and the synthesis of myofibrillar protein hits its peak [14]. The muscle then reaches a mature state with its residing progenitor cells, the SCs, acquiring a quiescent state in this tissue [11].

In somites, the high concentrations of FGF and Wnt in the caudal area lead to formation of mesenchymal cells in an undifferentiated state and this pathway also involves the control by Notch [15]. Then, the most dorsal part forms the dermomyotome, which will give rise to the majority of skeletal muscles. Cells of this compartment have high expression of the factors Pax3 and Pax7 and a low expression of the myogenic regulator Myf5 [16–18]. Afterwards, the maturation of a dermomyotome piece will form the myotome, which is characterized by the expression of MyoD and Myf5 [18–20]. Muscle progenitors subsequently intercalate into the primary myotome, and these will originate a fraction of the SCs that resides within the postnatal skeletal muscle [21–24].

**Figure 1:**
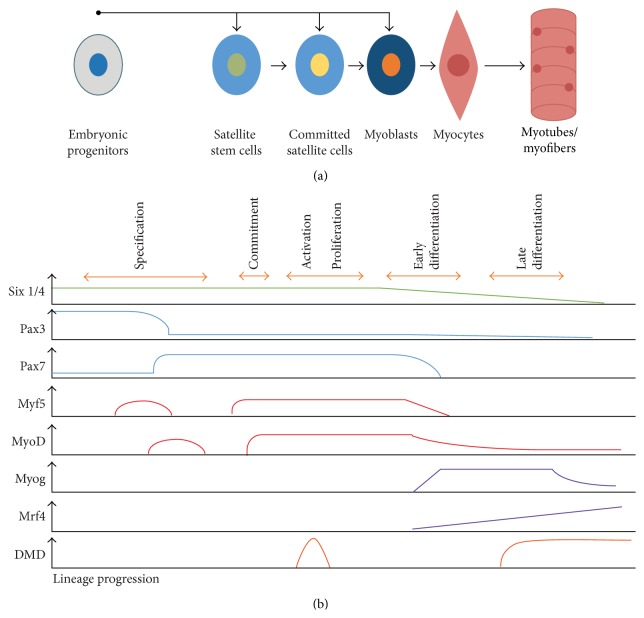
Cell hierarchy during development and adult myogenesis. (a) During development, an embryonic progenitor directly originates satellite stem cells, committed satellite cells, and myoblasts, which afterwards form a mature fiber. Some of them remain as satellite cells forming a heterogeneous population of stem and committed cells. In adult myogenesis, satellite cells can form myoblasts that will go through a similar process observed in development. (b) Genetic hierarchy of transcription factors involved in myogenesis. Six 1/4, Pax3, and Pax7 are the most important factors to muscle lineage specification, while Myf5 and MyoD prime cells to the myogenic program. Myog and Mrf4 control the myocytes fusion and the formation of myotubes. Dystrophin expression was recently also described in satellite cells. Adapted from Bentzinger et al., 2012 [11].

SCs are known to participate in adult muscle regeneration, and many similarities have been described between this process and the embryonic myogenesis, as relating SCs to progenitors of somatic origin [21–23, 25] (Figure 1(a)). It is also important to notice that the cells involved in the adult regeneration process are under the same genetic hierarchy involved in embryonic myogenesis, with the same genes participating in their regulation [26] (Figure 1(b)). The major distinction between myogenesis in the embryo and regeneration is that the latter requires a scaffolding that will work as a template [27].

A number of data also indicate that there are specific SCs that undergo asymmetric division, generating committed cells dedicated to the regeneration process, but also producing new SCs that are able to replenish the muscle stem cells pool [11].

Adult myogenic cells are derived mainly from SCs during late fetal development. However, there has been evidence of other adult stem cell populations that can also be involved in regeneration [12]. Nonetheless, it is remarkable that even though these other stem cells exist and have myogenic potential, experiments that deplete Pax7-satellite cells show that no other stem cell type is able to replenish the SC pool nor act in regeneration after injury, highlighting the unique importance of SCs [28].

## 3. Satellite Cell Markers

Satellite cells can be identified by the expression of several markers, with special attention to Pax7, which is considered the main defining factor for this cell type [5]. This marker has been correlated with the maintenance of an undifferentiated state, being an important factor for self-renewal in these cells [29]. In addition to Pax7, another protein from the paired domain transcription factor family might be expressed, Pax3, which is also important in the initial steps of muscle formation and is involved in the transcription of another marker, the tyrosine receptor kinase c-Met [30–32]. Interestingly, in the knockout mouse for Pax7, some SCs can be found, indicating that Pax3 alone could play a similar role [30, 33]. Conversely, other results suggest that Pax3 is not able to compensate for the Pax7 function [32]. In addition, the presence of Pax3 SCs is dependent on the muscle type [30].

**Figure 2:**
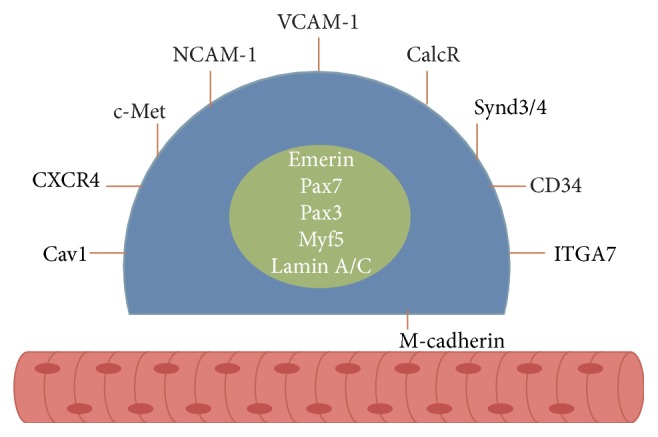
Principal satellite cell markers currently used for their identification.

Besides the Pax protein family, many other markers can be used to identify SCs such as the myogenic regulatory factor Myf5 [31, 34]; homeobox transcription factor Barx2, which is coexpressed with Pax7 and is a regulator of muscle growth, maintenance, and regeneration [35]; cell adhesion protein M-cadherin, which is known to be coexpressed with c-Met [31, 36]; cell surface attachment receptor 7-integrin [37, 38]; cluster of differentiation protein (CD34) that is expressed in quiescent SCs [34]; transmembrane heparan sulfate proteoglycans syndecan-3 and syndecan-4 [39]; chemokine receptor CXCR4 [40]; caveolae-forming protein caveolin-1 [38, 41]; calcitonin receptor, which was described as related to the quiescent state [38, 42]; vascular cell adhesion protein 1 VCAM-1 [43]; neural cell adhesion molecule 1 NCAM-1 [44, 45]; and nuclear envelop proteins lamin A/C and emerin [38]. However, these individual proteins are not exclusively expressed in SCs, meaning that only their simultaneous coexpression has been useful in identifying this cell type. Although other markers have been proposed to identify SCs, the ones cited above, and indicated in Figure 2, are the most commonly studied.  Table 1 is presenting examples of antibodies for immunofluorescence referred to in the literature. Different antibodies can be used according to the adopted methodology (western blotting, flow cytometry, etc.).

## 4. Heterogeneity in the Satellite Cell Population

Even though the identification of satellite cells is based on marker expression and morphological analysis, it has been suggested that these cells comprise a heterogeneous population of precursor cells [33]. It has also been reported that these cells can be prone to be committed either to the muscle lineage or to the self-renewal pathway, which is also an evidence of its heterogeneity [44, 46, 47]. The expression of the markers cited in the previous section, although well established in the literature, can be variable in this cell population, being another indication that this cell population can be heterogeneous, even though cells maintain their myogenic potential.

For instance, the expression of the marker Myf5 has been reported to be absent in ~10% of the SC population, and the cells identified as Pax7+/Myf5− contributed to their reservoir in contrast with Pax7+/Myf5+ cells that were committed to differentiation [48]. Studies also showed that activated cells expressing low levels of Pax7 were more committed to differentiation, whereas high levels of Pax7 were related to cells less prone to differentiate and that had more undifferentiated characteristics [49]. Experiments with histone 2B labeling also demonstrated that there are SCs that retain or lose this mark and that the former is able to self-renew and the cells that lose the mark are restricted to differentiation [50].

Differences were also observed in the proliferation rate of SCs, as slow and fast dividing cells coexist [51]. The slow ones are capable of long-term self-renewal, whereas fast dividing cells compromise themselves with the myogenic lineage without producing self-renewing progeny [52]. In this sense, subpopulations that are considered committed to the myogenic lineage could participate in the regeneration of an injured muscle before the ones that are still in the more progenitor state and so would take a longer time to be involved in this process [53]. This scenario is consistent with a stem cell to progenitor cell hierarchy.

As it is known that muscles within the body are distinct between themselves, it has been seen that SCs also present heterogeneity based on the muscle they are located within, which may correlate to their distinct embryonic origin [6, 54]. This is consistent with the previous results observed by Buckingham et al. and Relaix et al. that shows that the expression of Pax3 by SCs is muscle-dependent [30, 32]. As knowledge increased, studies were done to determine whether the heterogeneity of SCs in muscle was due to the muscle environment or internal programming, and the outcomes of distinct researches showed that there is evidence for both [55, 56].

Differences in SCs were also found when considering the extrafusal fibers and their categorization into fast and slow fibers regarding proliferation rate and differentiation potential. Remarkably, the SCs could differentiate into exclusive fast fibers when they came from a fast muscle and into fast or slow fibers when they are derived from a slow muscle [57–59]. As observed previously, the phenotype after differentiation can either be dependent on the intrinsic programming that is related to the fiber type or can be under influence of the environment, that is, the muscle fiber with whom the cell interacts [6, 60]. As for intrafusal fibers, SCs in this compartment are known to be more plastic and directed to a specific phenotype by foreign innervation stimulation [61, 62].

Morphological differences translated as round and thick cells were also observed in the SC population and they were associated with distinct myogenic potential [63], the thick ones being more prone to differentiation. Functionally, there are observations that suggest two subpopulations of SCs, one that is committed to muscle growth, whose cell number declines with age and is present in a larger amount in males, and another subpopulation related to muscle regeneration after an injury, whose cell amount is relatively maintained during aging and is not gender related [64].

The heterogeneity may also result from the distinct niche in which these cells are located, as has been observed in the aging process, where cells may escape quiescence and lose their capacity of self-renewal [65–67]. An important component of the muscle niche that acts directly in proliferation and differentiation of satellite cells is the fibro/adipogenic precursors, and it is known that they act positively in young* Dmd* ^*mdx*^ mice, the model for Duchenne Muscular Dystrophy, but repress the formation of myotubes in old ones, indicating that the process of aging has direct implications in satellite cells [68]. Other factors such as Notch and Wnt are also involved in this nonautonomous process of SCs aging [67, 69]. In addition, intrinsic changes in cells are also observed in the aging process, such as in geriatric SCs that lose the reversible quiescence and enter in a presenescence that cannot be reversed and that in an injured muscle fail to start the regenerative process and enter in a full senescence state [70]. It was also shown that intrinsic cell factors also lead to the loss of self-renewal with the involvement of the MAPK pathway [71, 72]. It is important to distinguish between autonomous and cell nonautonomous factors that interfere with SCs in aging, since the nonautonomous ones, such as the niche, can be corrected with a youthful environment [73], a fact that cannot be corrected when the factor is intrinsic to the cell [70].

This heterogeneity in the stem cell population in muscle has been complicating the identification, function, and naming of these cells. There has been in the literature a description of other types of cells with high myogenic capacity and directly related to muscle regeneration, called muscle derived stem cells that express distinct markers [74]. Nonetheless, it is important to notice that there have been subsequent results indicating that, without SCs, no other cell types have the capacity to regenerate muscle [28]. This may be either because the other cell types studied did not include the specific population described by Qu-Petersen and colleagues [74], or that the activity of other cell types has a requirement for use in conjunction with SCs or with the major SC factor Pax7 [75].

Additionally, diverse muscle derived stem cell (MDSC) populations had been identified. These populations include myogenic progenitor cells characterized as CD56+, CD34−, CD144−, CD45−, and CD146−; CD56+, CD34+, CD144+, CD45−, and CD146− mio-endothelial cells; CD56−, CD34−, CD144−, CD45−, and CD146+ perivascular progenitor cells; and a muscle derived side population which has similar features to bone marrow stem cells [76]. Based on adhesion and proliferative properties, Qu-Petersen and colleagues [74] isolated three cell populations derived from muscle. Two of these populations, EP (early preplate) and LP (late preplate), represent the satellite cells; the third one, which also adheres lately, is named MDSC and presents characteristics usually associated with noncommitted progenitor cells. The EP population represents the majority of the cells obtained from muscle digestion and differentiates into myotubes. However, EP cells have a limited regenerative potential. The LP population accounts for about 1% of satellite cells, but it has low rates of proliferation and differentiation. Conversely, MDSC showed a better self-renewal ability and sustained proliferation and are multipotent. Thus, the MDSC would be less committed cells and more promising for therapies in comparison to satellite cells [74].

Other cell types, such as bone marrow mesenchymal stem cells [77–81], adipose derived mesenchymal stem cells [82–84], CD133+ cells [85–87], pericytes/mesoangioblasts [88, 89], and side population cells [90, 91], were described as being able to participate in myotube formation as well as replenishing the satellite pool. These cells are not initially committed to muscle and may not express the classical satellite cell marker, Pax7. However, they are capable of contributing to muscle regeneration when fusing with myogenic cells and, additionally, they may be able to turn into Pax7 expressing cells originating new SCs, which is a fact that may strongly contribute to the heterogeneity observed in this population. It is also important to notice that evidence has been found that myogenic cells are formed by fusion [78, 92–94] or transdifferentiation, in which cells develop into intrinsically myogenic ones [95–97], and the heterogeneity would rise by the contribution of both cells that participate in the fusion process or by one cell initially not committed to muscle becoming myogenic. Furthermore, other cell types may be involved in assisting muscle regeneration sending signals that direct differentiation of SCs, such as fibro/adipogenic precursors [98–100]. It is clear then that whether one cell type turns into muscle by fusion or transdifferentiation or that the precursor itself receives signals that direct their proliferation and differentiation, the final outcome is that all these factors contribute to the myogenic population being heterogeneous.

## 5. Satellite Cells Can Undergo Multilineage Differentiation

Besides their myogenic potential, it has been described in the literature that these cells can undergo osteogenic and adipogenic differentiation, for example. This highlights their properties as a stem cell that is able to differentiate within the mesenchymal lineage [101–104].

Studies in rat showed that the heterogeneity in the proliferation rate correlates with the differentiation potential, with high proliferative clones being able to differentiate into adipocytes [105]. Morphological heterogeneity was also related to distinct potential, with thick cells also being able to undergo osteogenic differentiation [63]. Heterogeneity also in the CD34 expression was correlated with distinct potential to go through the adipogenic pathway, and only cells that expressed this marker were able to undergo adipogenic differentiation [106].

Additionally, in aged mice, it was observed that SCs tend to go to the fibrogenic lineage instead of maintaining their myogenic potential, which may contribute to the greater fibrosis observed in old mice [69].

## 6. The Balance between Quiescence and Activation

Skeletal muscle regeneration follows a series of steps that recapitulates the phases of development. First, muscle progenitor cells must exit the state of quiescence and become active and proliferate. Asymmetric divisions are important to provide daughter cells committed to the myogenic program (myoblasts) and also daughter cells that return to quiescent state in order to replenish the stem cell pool. After proliferation, myoblasts differentiate and fuse to form myotubes, which fuse with each other or to a previous fiber to repair it. Finally, the myofibers grow and maturate.

### 6.1. Quiescence Mechanisms

**Figure 3:**
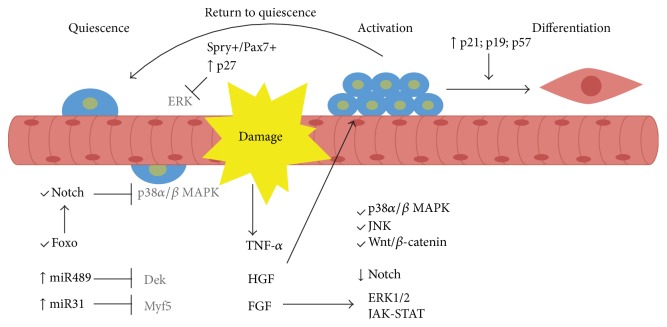
Quiescence and activation control. Notch signaling is one of the main pathways controlling quiescence. Foxo transcription factors regulate Notch receptors expression facilitating this pathway's activity. MicroRNAs target cell cycle genes and myogenic regulators. Upon injury, signaling molecules are released and pathways related to cell cycle progression are turned on. After some rounds of division, some cells return to quiescence whereas the others exit cell cycle and proceed into the myogenic program, differentiate, and repair the damage.

As other types of adult stem cells, SCs are quiescent until they are activated when there is a muscle injury. Maintenance of quiescence is crucial to preserve the SC pool and it is controlled by different molecular mechanisms, with participation of many genes and regulatory pathways. Microarray studies showed that more than 500 genes are overexpressed in quiescent SCs in comparison with proliferating myoblasts [42]. Negative regulators of cell cycle are among these genes. Despite the fact that all the players and mechanisms of SCs' homeostasis are not being fully understood, many efforts have been employed in order to depict them (Figure 3).

The Notch signaling was implicated in SC quiescence maintenance, as well as proliferation and differentiation regulation, in various studies [107–111]. Indeed, Notch signaling was established as the first quiescence regulator in adult stem cells because an interruption in Notch activity favors spontaneous cell differentiation, without the entry in the S phase [110]. The highest activity of Notch signaling is seen in quiescent SCs and it is progressively reduced as the cell progresses through myogenic differentiation. Interestingly, Notch signaling prolonged blockage does not prevent cells from proliferating but leads to depletion of SC, demonstrating that it is necessary for self-renewal [110]. A similar study showed related results about the loss of Notch signaling by RBP-J deletion. The absence of Notch signaling has at least three main effects: failure of quiescence maintenance; loss of the ability to self-renew; and spontaneous differentiation, without a phase of proliferation [108].

The FOXO family of transcription factors regulates stem cell pools in adult tissues. The levels of* Foxo3* transcript and protein are higher in quiescent SCs than in activated ones. The ablation of* Foxo3* gene specifically in SCs showed that this transcription factor is important for SC return to quiescence and self-renew. FOXO3 negative cells are more proliferative and differentiate more rapidly, while* Foxo3* overexpression suppresses cell cycle entry and represses terminal differentiation [112]. This work also links FOXO3 to Notch signaling: FOXO3 regulates NOTCH1 and NOTCH3 receptor expression, activating Notch signaling, and thus promotes quiescence in SCs [112].

MicroRNAs are significant players in gene expression regulation, including genes related to stem cell functions; and their activity in SCs regulation has been recently explored. It was demonstrated that miR-489 is highly expressed in quiescent SCs and is downregulated as they become activated. A target of miR-489 is* Dek*, an oncogene, whose both mRNA and protein levels are higher in activated SCs than in quiescent SCs. In SCs, Dek promotes proliferation after activation; Dek-positive cells are committed to myogenic differentiation and Dek-negative cells are self-renewing [113]. Another miRNA involved in SCs quiescence is miR-31. Although the majority of SCs in adult tissue have the* Myf5* gene activated [48], they do not necessarily differentiate, which implies that a mechanism must exist to prevent* Myf5* mRNA translation before the appropriated moment. This repression is accomplished by miR-31 that has a higher expression in quiescent SCs; it targets* Myf5* mRNA and then sequesters it in mRNP granules. Upon activation, miR-31 levels decrease and* Myf5* mRNA is released to translation [114].

SCs quiescence is also established by mRNA decay. Hausburg and colleagues showed that* Myod* transcript is driven to mRNA decay, preventing the SC to proceed in the myogenic program. This is achieved by the action of the protein tristetraprolin (TTP) that binds to mRNA, preventing it to be translated and, in addition, regulating its decay [115].

All these posttranscriptional regulation mechanisms seem to be somewhat redundant and they seem to act in a subpopulation-specific manner; however, more studies are necessary to clarify all the mechanisms involved in quiescence maintenance and to define whether they are common to all SCs.

### 6.2. Activation and Proliferation Mechanisms

When the muscle suffers an injury, the SCs must be activated, starting to proliferate and differentiate to repair and/or form new muscle fibers. SC activation is a transient process regulated at different levels. As Notch inhibits p38*α*/*β* MAPK signaling pathway in quiescent stage [116], this is the first pathway to be activated [117], resulting in the expression of* Myod *and consequent cell cycle entry. The damaged fibers release many growth factors that induce the activation of signaling pathways related to cell cycle, like TNF-*α*, HGF, and FGF [118–120]. The transition from G1 to S phase is achieved by activation of ERK1/2 pathway by Fgf2 [121]. Another MAPK signaling pathway involved in SC cell cycle progression is JNK [122].

The intense cell proliferation is important to muscle repair, but it has to be limited and the fate of each daughter cell must be determined—terminally differentiate or return to quiescence. Wnt/*β*-catenin signaling is temporally activated during regeneration but later downregulated to limit the regenerative response [123]. Wnt/*β*-catenin signaling is also involved in the promotion of myogenic differentiation. The treatment of SCs with Wnt3a promotes cell cycle arrest, myogenin activation, and follistatin expression, promoting myoblast fusion and terminal differentiation [124].

The JAK-STAT signaling is another player in the regulation of SC function, especially in the aged muscle, whereby Stat3 activation interferes in MyoD to promote myoblast differentiation [125]. JAK-STAT signaling increases progressively with age or disease. Jak2 and Stat3 transient inhibition in aged and dystrophic muscle enhances SC expansion and better muscle regeneration [126, 127].

### 6.3. Cell Cycle Exit

To exit the cell cycle, upregulation of inhibitors of cyclin-dependent kinases is required. The return to quiescence requires p27^kip1^, whereas the progression through myogenesis requires the upregulation of p21^Cip1^, p19^Arf^, and p57 [50, 128, 129]. Sprouty1 (*Spry1*) is a receptor tyrosine kinase signaling inhibitor expressed in Pax7^+^ quiescent cells, but downregulated in proliferating myoblasts. When Pax7^+^ cells return to quiescence Spry1 is induced again, promoting cell cycle exit by inhibiting ERK pathway [130].

### 6.4. Asymmetric Divisions and Self-Renewal

The daughter cells asymmetry, that is, segregation of different determinant factors, will determine whether they differentiate or self-renew. The myogenic determinant factors Myf5, MyoD, and Myog have asymmetric expression in the daughter cells [48, 131, 132]. MyoD is distributed to committed Pax7− cells and the Pax+/MyoD− cells are self-renewing [133]. For Myog, the same is observed: the myogenic lineage is Pax7−/Myog+ and reservoir cells are Pax7+/Myog− [134]. The distribution of DNA template is also asymmetric: the old template goes to the daughter cell expressing Pax7, the reservoir cell, and the new DNA template to the one expressing Myog [134].

In Myf5-negative SCs, those compromised with renovation of the stem cell pool, the Notch3 receptor is enriched, whereas Myf5-positive cells receive the Notch ligand Delta1 [48]; in Myog-positive cells there is also the presence of Numb, a Notch antagonist [107]. All these findings are related to the role of Notch signaling in maintenance of quiescence, as discussed above.

## 7. Satellite Cells in the Context of the Muscular Dystrophies

Different hypothesis and mechanisms are proposed to explain the muscular degeneration that occurs in patients bearing mutations in a wide number of genes important to muscle structure and function [135, 136]. As the dystrophic muscle is persistently injured, the regenerative process is consistently activated, recruiting satellite cells at higher rates than in normal tissue. Nevertheless, in dystrophic muscle, the regeneration is not complete and there is a progressive replacement of muscle by fibrofatty tissue. Thus, the ability of stem cells to repair the muscle is not sufficient to compensate for degeneration. Three scenarios are proposed to explain this limited regenerative capacity [135].

First, the repetitive cycles of replication would lead SCs to senescence, due to telomere shortening. The presence of shortened telomeres was observed in DMD (Duchenne Muscular Dystrophy) and LGMD2C (limb-girdle muscular dystrophy type 2C) patients [137, 138] and in* Dmd* ^*mdx*^ mice [139].* Dmd* ^*mdx*^ mice lacking telomerase activity develop a phenotype more faithful to muscular dystrophy in humans, including a worsening with aging [140]. However this is controversial, as another study could not detect a significant telomere shortening [141].

Second, the differentiation could not be adequate. Early studies showed that myoblasts from DMD patients delay to fuse and present an abnormal differentiation [142, 143]. In some types of muscular dystrophy, the mutated gene is not expressed in SCs and thus does not influence directly on their function [144]. However, there is also evidence that the primary mutation itself can impair the SC function by reducing its number and causing premature senescence, implicating SC as directly involved in the disease mechanism [145]. Alterations in signaling pathways are also underlying the regenerative potential of SCs. In a knock-in conditional mouse in which Notch signaling is blocked in SCs, the muscle develops a typical dystrophic phenotype with impaired regeneration [146]. The SCs of this mouse showed reduced activation and proliferation, but enhanced differentiation, corroborating the previous studies about the role of Notch signaling in quiescence maintenance [146]. In* Dmd* ^*mdx*^ mouse, the Notch signaling is attenuated, which diminishes SCs self-renewal; and the constitutive activation of Notch recovered the self-renewal capacity, but this is not sufficient to improve regeneration, probably because of MyoD and myogenin inhibition [111].

Dystrophin is expressed in differentiated myofibers, but not in proliferating myoblasts; thus it was believed that it was not expressed in satellite cells either. However, a recently published paper elegantly showed that dystrophin is indeed expressed by satellite cells and that it plays an essential role in the regulation of their polarity and asymmetric division. In the absence of dystrophin, there is a reduction in the number of asymmetric divisions and more abnormal divisions, which lead to a decrease in the quantity of myogenic progenitors and thus a failure in muscle regeneration [147]. This work adds a major role for satellite cells dysfunction in the pathophysiology of DMD, which has direct implications for therapies. Third, the dystrophic niche is not favorable for regeneration. In the dystroglycanopathy mouse model* Large* ^*myd*^, an increased number of SCs were found in freshly isolated single fibers, related to control mouse [148]. As long as SCs remained attached to the fibers, their proliferative capacity was seen to be reduced, but after total isolation they proliferated and differentiated at levels comparable to normal control, indicating an important role of the niche to stem cell function [148]. In this mouse model, the basal lamina composed by an excess of fibronectin and collagen acts as an obstacle to proper SC proliferation. This work contradicts a former one which suggested that as SC also expresses dystroglycan, the glycosylation defect would also affect its function, impairing regeneration [149]. Even though a recent publication reinforces that the regenerative capacity is not affected in muscles with glycosylation deficiency, the inability to overcome the degeneration is more related to the depletion of regenerative capacity due to excessive and progressive degeneration that occurs in muscular dystrophies than to an inherent defect in SC function itself [150].

By testing the effects of irradiation and myotoxins in the engraftment of donor SCs in nude* Dmd* ^*mdx *^mouse it was found that when the host SC pool is still preserved, the engraftment is poor; in contrast, when the host SC pool is incapacitated by irradiation, but the stem cell niche is preserved, the donor cell is able to repopulate and regenerate the muscle [151]. Boldrin et al. investigated the regenerative potential of SCs isolated from young and old* Dmd* ^*mdx*^ mice. They found that both young and aged SCs are able to regenerate the muscle of preirradiated young nude* Dmd* ^*mdx*^, reinforcing the notion that the SC function is preserved and that the dystrophic environment, instead of an inherent defect, influences it negatively [152]. The main message of these works is that for future cell therapies it will be interesting to capacitate the host stem cell pool, as well as the preservation/amelioration of a functional niche, to obtain successful results.

An important study performed in several animal models also gave an insight into how the satellite cells are regulated in a context of muscular dystrophies [153]. In the* SJL/L* mouse model for the limb-girdle muscular dystrophy type 2B the levels of* MyoD* and* Myf5* were found to be downregulated, which indicates that in this animal the satellite cells remain quiescent, which is expected since the histopathology of this animal shows no evidence of the degeneration and regeneration process. This same downregulation was found in the animal* Large* ^*myd*^, which is consistent with previous results that shows that the mutation in this animal could interfere with the satellite cell functioning and self-renewal [149]. On the other hand, the animal models* Dmd* ^*mdx*^ and* Lama2* ^*dy-2J*^ */J, *the models for congenital muscular dystrophy type 1A, showed enhanced expression levels of* MyoD* and* Myf5*, indicating that in these models the satellite cells are activated, which is consistent with the presence of regeneration areas in their histology.

## 8. Therapies

Since the identification of stem cells, the most promising therapy for muscle wasting diseases has been the cell therapy. The first myoblast transplantation was done in the late 1970s when it was shown that donor myoblasts were able to fuse within host myofibers [154]. One decade later, the demonstration that donor myoblasts restored dystrophin expression in* Dmd* ^*mdx*^ myofibers [155] opened the precedent for many human clinical trials [156–163]; nevertheless, the results were not satisfactory, mainly by the reduced regenerative potential of myoblasts, once they are more committed and differentiated in comparison to SCs.

Entire myofibers can be grafted into host muscle where SCs attached to donor myofibers contribute to muscle regeneration [46]. The advantages of myofiber transplant are as follows: a maximal engraftment is required, a minimal number of cells are required, and the cells are transplanted together with their niche, although these are not easy to apply in clinics [164].

SCs isolated by flow cytometry were transplanted in* Dmd* ^*mdx*^ mice and it was seen that they engrafted into their muscles and also contributed to the SC compartment, but if the cells were cultured before transplantation, their regenerative potential was reduced [165]. The transplantation of a single luciferase-expressing SC helped to verify the fact that it can self-renew and differentiate, demonstrating the relevance of a careful selection of which cell to use given the high population heterogeneity [166]. Taken together, the studies about direct isolation and transplantation of SCs show the advantages of the requirement of a low number of cells, efficient engraftment, and the repopulation of the host niche with new SCs; in contrast, the migration of transplanted cells is limited, only a small number of cells are isolated, and they cannot be maintained for a long time in vitro [164].

Therefore, the use of progenitor cells like SCs is more promising with the advantage of also replenishing the stem cell pool with the possibility of a sustained response. However, the use of these cells in therapy is still not a reality and many challenges remain to be overcome. These include selection of the most suitable subpopulation, optimal culture conditions, and modulation of signaling pathways that control quiescence and self-renewal and delivery of the cells. The choice between systemic and local injections must consider specific features of each disease, like disease severity and the number and size of affected muscles. Still, both strategies have their limitations and issues including homing, engraftment, and long-term survival. Thus, given all the aspects to be dealt and the divergence between in vitro and in vivo results, the combination of different strategies would be more promising.

## 9. Conclusion

Satellite cells are the first in line for muscle regeneration, and so they are the most promising target in a cell-based therapy for muscle wasting disorders. As shown throughout this review, they have numerous advantages such as easy identification, self-renewal, and myogenic differentiation, which is well understood, and they have been already tested in a therapeutic context. Nevertheless, many questions remain to be answered and this review aimed to explore some possible aspects that could be considered in order to achieve an efficient cell therapy.

At first, the heterogeneity of this population should be considered, such as choosing the ones with better capacity of self-renewal to replenish the pool in an injured muscle or the ones that could be more prone to differentiation. Additionally, since SCs from different muscles or fibers can be distinct, it is important to consider these aspects in order to treat a specific muscle group, for example. The quiescence and activation process is also an aspect that should be considered, since it can be regulated and used, for instance, to direct activation of resident cells. Finally, with previous studies regarding muscular dystrophies and therapies, it is possible to learn about ideal culture conditions and better ways to deliver cells, for example.

It is important to notice that an issue regarding nomenclature of different types of satellite cells may complicate the data interpretation and comparison across studies, since different terms are sometimes used by authors for the same type of cells, or different cells are referred to them with the same general nomenclature. It is possible, therefore, that authors are dealing with the same entities but naming them differently. Hence, it would be valuable if the scientific community found a consensus concerning the diversity of the various cell populations studied.

Major hurdles still have to be dealt with, such as the wide distribution of skeletal muscles within the body and the effect of genetic defects in resident cells; however, this review proposes that the knowledge of the satellite cells basic biology may help in the development of further cell-based therapies.

## Figures and Tables

**Table 1:** Examples of antibodies used to identify satellite cells by immunofluorescence. Hu = human; Mo = mouse.

Protein	Company/catalogue number	Reacts with	Reference
Pax7	Hybridoma Bank (DSHB)	Hu/Mo	Dumont et al., 2015 [147]

MyoD CXCR4	Santa Cruz Biotechnology/C-20 Abcam/not mentioned	Hu/Mo Hu/Mo	Cerletti et al., 2008 [167]

Barx2	Santa Cruz Biotechnology/sc-9128	Hu/Mo	Meech et al., 2012 [35]

Syndecans 3 and 4	Non-commercial antibody	Mo	Cornelison et al., 2001 [39]

M-cadherin	BD Biosciences/611101	Mo	Marti et al., 2013 [168]

Caveolin-1	Santa Cruz Biotechnology/sc-894	Hu/Mo	Gnocchi et al., 2009 [38]

CD56/NCAM	BD Biosciences/347740	Hu	Lindström et al., 2015 [169]

Pax3	Hybridoma Bank (DSHB)	Mo	Kirkpatrick et al., 2010 [170]

c-Met	Novocastra Laboratories/CMET-S	Hu	Lindström et al., 2010 [171]

CD34	PharMingen/clone RAM34	Mo	Beauchamp et al., 2000 [34]

Myf5	Santa Cruz Biotechnology	Hu/Mo	Günther et al., 2013 [172]

Calcitonin receptor	AbD Serotec/AHP635	Hu/Mo	Yamaguchi et al., 2015 [173]

Desmin Lamin A/C	DAKO/clone D33 Cell Signaling/2032	Hu/Mo Hu/Mo	Frock et al., 2006 [174]
